# Mild Hypobaric Hypoxia Enhances Post-exercise Vascular Responses in Young Male Runners

**DOI:** 10.3389/fphys.2019.00546

**Published:** 2019-05-24

**Authors:** Yoko Saito, Mariko Nakamura, Kazumi Eguchi, Takeshi Otsuki

**Affiliations:** ^1^Faculty of Human Sciences, Kanazawa Seiryo University, Kanazawa, Japan; ^2^Department of Sports Sciences, Japan Institute of Sports Sciences, Tokyo, Japan; ^3^Faculty of Sport and Health Sciences, Ryutsu Keizai University, Ryugasaki, Japan

**Keywords:** athletes, maximal exercise, mild hypobaric hypoxia, vascular resistance, post-exercise vasodilation

## Abstract

It has been reported that sustained post-exercise vasodilation may be linked to exercise-induced angiogenesis. The present study aimed to evaluate whether mild hypobaric hypoxia enhances the post-exercise reduction in systemic vascular resistance in young male runners. Seven male intercollegiate runners (aged 19–21 years) performed maximal incremental treadmill running under conditions of hypobaric hypoxia (corresponding to 2,200 m above sea level, hereinafter referred to as HH) and normobaric normoxia (corresponding to sea level, hereinafter referred to as NN). A third exercise test was performed under NN conditions, consisting of submaximal exercise with the same absolute exercise volume as was achieved during HH (submaximal exercise under NN conditions, hereinafter referred to as NNsubmax). Blood pressure and cardiac output (CO) were measured before and at 15, 30, and 60 (p60) minutes after exercise. Compared with NN, exercise time was shorter in HH and NNsubmax conditions (*p* < 0.05). Systolic blood pressure and mean blood pressure (MBP) were lower after exercise in HH conditions (*p* < 0.05). No condition-related differences were found in CO. Total peripheral resistance (TPR, defined as the ratio of MBP to CO) was significantly lower after exercise compared to baseline for all conditions (*p* < 0.05). However, the decrease in TPR was maintained longer after exercise in HH compared with NN and NNsubmax conditions (*p* < 0.05). At p60, TPR was lower than baseline for HH conditions (*p* < 0.05), whereas after exercise in NN, and NNsubmax conditions, TPR recovered to baseline by p60. Decreases in systemic vascular resistance after exercise were maintained longer under mild HH conditions compared with NN despite the lower exercise volume of the former.

## Introduction

During recovery from exercise, dynamic changes in neural, and local factors induce immediate hyperemia (0–20 min after exercise) and sustained vasodilation (20–120 min after exercise) (Laughlin et al., 2012). Halliwill et al. (2013) proposed that sustained vasodilation after exercise may contribute to the growth and remodeling of microvasculature. Increased capillary supply improves the delivery of oxygenated blood to working muscles, and thus increases the arteriovenous oxygen differences by increasing the diffusion area within tissues. These changes lead to increased oxygen uptake and consequent improved performance in endurance exercise (Brodal et al., 1977). Therefore, establishing a method which can prolong or enhance sustained post-exercise vasodilation may be beneficial for endurance athletes who require maximal aerobic capacity.

Altitude training is a popular method used by competitive athletes, because hematological mechanisms (changes in red blood cell mass), and non-hematological mechanisms (such as angiogenesis, glucose transport, glycolysis, and PH regulation) of adaptation to hypoxia may enhance competitive performance (Gore et al., 2007; Girard et al., 2017). Additionally, combining exposure to hypoxia with exercise has been reported to improve O_2_ transport and/or metabolism within muscles (Lundby et al., 2009), suggesting that exercise under such conditions may induce angiogenesis. If angiogenesis is in fact induced by post-exercise vasodilation, then exercise-induced vasodilation might be enhanced at higher altitudes. Indeed, inspiration of hypoxic gas has been shown to increase vascular conductance during submaximal exercise via the endothelial function-related pathway, as described by Casey et al. (2010), and enhance flow-mediated dilation (FMD) after submaximal exercise (Katayama et al., 2013) in young sedentary males. However, hemodynamics after intense (maximal and supramaximal) endurance exercise under conditions of hypobaric hypoxia have not been explored. It has been reported that maximal exercise under normoxic conditions causes a transient increase in reactive hyperemia (Schroeder et al., 2019), and that high-intensity interval training under hypoxic conditions improves maximal oxygen uptake and performance during repeated-sprint exercise (Brocherie et al., 2017). Studies on endurance athletes could have clinical implications for other athletes because the effects of exercise and altitude may differ between trained and sedentary individuals.

The accompanying negative effects of altitude training must be considered along with the training benefits. The incidence and severity of acute mountain sickness symptoms such as headaches, dizziness, fatigue, and restless sleep depend on the altitude reached and rate of ascent (Hackett et al., 1976). The hypoxic conditions in the studies mentioned above (Casey et al., 2010; Katayama et al., 2013) were relatively severe, at <80% arterial oxygen saturation (SpO_2_); in terms of safety, studies in a milder hypoxic environment are important. To the best of our knowledge, there have been no investigations into the effects of exercise under mild hypoxic conditions on sustained post-exercise vasodilation.

We hypothesized that mild hypobaric hypoxia can enhance sustained post-exercise vasodilation in endurance athletes. To test this hypothesis, we evaluated the effects of artificial altitude conditions equivalent to 2,200 m above sea level on the systemic vascular resistance after maximal endurance exercise in young male runners. We chose 2,200 m because the arterial O_2_ saturation reduced at this altitude may be sufficient to stimulate vasodilation after exercise, as indicated by a previous study which reported that 8 weeks of aerobic training under similar hypobaric hypoxic conditions improved FMD, and arterial stiffness in postmenopausal women (Nishiwaki et al., 2011).

## Materials and Methods

### Subjects

Seven male intercollegiate long-distance runners were recruited for this study [age (mean ± standard deviation) 19.9 ± 0.9 years; height, 173.0 ± 4.3 cm; weight, 57.8 ± 6.2 kg]. None of the subjects had cardiovascular disease, including hypertension.

A power calculation was performed to calculate adequate sample size for repeated measures two-way analysis of variance (ANOVA) analysis of post-exercise changes in systolic blood pressure (SBP). We used the G^∗^Power 3 program (Faul et al., 2007). The sample size of this study (*n* = 7) was adequate to detect an interaction at 80% power with an α value of 5% when effect size was assumed as the medium (0.25) (Cohen, 1992).

All subjects gave written informed consent after verbal explanation of this study and the anticipated risks, in accordance with the Declaration of Helsinki. The study and all its procedures were carried out in accordance with the recommendations of, and approved by, the Ethics Committee of the Japan Institute of Sports Sciences.

### Procedure

Trials were conducted under conditions of normobaric normoxia (760 mmHg, corresponding to sea level, hereinafter denoted NN) and hypobaric hypoxia (568 mmHg, corresponding to 2,200 m above sea level, hereinafter denoted HH) in a hypobaric chamber. In our pilot study, SpO_2_ was approximately 10% lower in HH compared with NN conditions, and HH conditions resulted in decreased exercise time. Thus, as the third condition, submaximal exercise was performed with the same absolute workload as in HH conditions, under NN conditions (hereinafter denoted NNsubmax, 760 mmHg). This aimed to compare the effects of exercise of equivalent workload in hypoxic vs. normoxic conditions. Subjects underwent NN and HH trials in a randomized order. The NNsubmax trial was conducted after the HH trial, or after both the HH and NN trials.

Subjects visited the chamber four times over a period of 3–16 days. During the first visit, we explained the study in detail and the subjects became accustomed to using the experimental instruments. Data collection was carried out during the other three visits. Changes in cardiovascular status for the three different conditions were measured during a 60-min recovery period after acute exercise. Subjects fasted for 12 h prior to each test and were instructed to avoid strenuous activity and caffeine for 24 h prior to each test.

### Exercise

#### Exercise Mode

The exercise test consisted of running on a treadmill (Biomill, 4 Assist, Tokyo, Japan). Subjects warmed up on the treadmill for 10 min while heart rate (HR) was monitored with an electrocardiographic signal transmitter (ZS-910P, Nihon Kohden, Tokyo, Japan). Running speed was self-paced and therefore not defined. Next, the maximal incremental running test was performed. The test began with the treadmill speed set at 286 m/min and the speed was increased by 14–20 m/min every 3 min in the first four stages. Starting at the fifth stage, the speed was increased by 11 m/min every 3 min. In the NN and HH trials, the test continued until subjects became exhausted. For the NNsubmax trials, the trial ended when subjects reached the time that was attained in the previous HH trial.

The exercise test was discontinued if three or more of the following five criteria were met: (1) a rating of perceived exertion greater than 17 on the Borg Scale, (2) a respiratory exchange ratio greater than 1.1, (3) no increase in HR with increasing running speed, (4) plateaued oxygen uptake (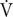
O_2_) (increase of 150 mL or less) with increasing running speed, and (5) subject request for discontinuation due to fatigue.

#### Exercise-Related Measurements

Ventilatory parameters and the oxygen (O_2_) and carbon dioxide (CO_2_) concentrations of expired air were analyzed breath-by-breath during the running test using an open-circuit spirometry gas analysis system (AS300, Minato Medical Science, Osaka, Japan). The system was calibrated prior to measurements under NN and HH conditions with a calibration gas of known O_2_ and CO_2_ concentration and constant volume.

Mean minute ventilation (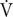
E), 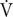
O_2_, CO_2_ production (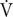
CO_2_), and HR were calculated for every 30 s of exercise. The mean values of 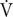
E, 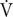
O_2_, and 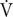
CO_2_ during exercise were defined as 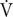
Emean, 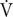
O_2_mean, and 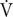
CO_2_mean, respectively. Peak 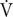
O_2_ and HR were defined as 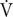
O_2_peak and HRpeak, respectively.

### Post-exercise

#### Rest and Recovery

The air temperature in the chamber was maintained between 23 and 25°C. Baseline measurements of cardiovascular parameters were taken after the subjects had rested for at least 15 min in the supine position. Five minutes after the completion of the exercise, the subjects were instructed to rest in the supine position for 55 min (i.e., to reach 60 min after exercise), and the cardiovascular status was measured at 15 (p15), 30 (p30), and 60 (p60) minutes after cessation of exercise. Subjects were allowed to drink water *ad libitum* during the experimental period.

#### Cardiovascular Measurements

##### Oxygen saturation

Measurements of SpO_2_ were performed using a pulse oximeter (OLV-3100, Nihon Kohden, Tokyo, Japan) placed on the tip of the left index finger before exercise.

##### Arterial blood pressure and heart rate

Diastolic blood pressure (DBP), SBP, and HR were each measured three times at the upper right arm using the oscillometric method (Jentow, Nihon Colin, Aichi, Japan). The mean values of the three measurements of each parameter were used for analysis. Mean blood pressure (MBP) was estimated using the following formula: MBP = 2/3(DBP) + 1/3(SBP).

##### Cardiac function

Echocardiography was performed (SSD-6500, Aloka Company, Tokyo, Japan) according to the recommendations of the American Institute of Ultrasound in Medicine (Sahn et al., 1978). First, B-mode long axis views of the left ventricle from the left parasternal region were obtained with a 1.88-MHz sector probe. Next, M-mode measurements were made over 10 continuous heartbeats. Echocardiographic video images were converted from analog to digital information (Mini Converter, Blackmagic Design, Fremont, CA, United States), exported, and stored in a general-purpose computer. The images were analyzed offline using image-processing software (ImageJ, National Institute of Health, Bethesda, MD, United States). Left ventricular end-diastolic diameter and left ventricular end-systolic diameter were measured, and the mean values over three heartbeats calculated. End-diastolic volume and end-systolic volume were calculated using the method of Teichholz et al. (1976). Stroke volume was obtained by subtracting left ventricular end-systolic volume from left ventricular end-diastolic volume. CO was calculated as the product of HR and stroke volume. Total peripheral resistance (TPR) was calculated using the following formula: TPR = MBP/CO. The between-day coefficient of variation for stroke volume was calculated to be 6.8% in the present study.

##### Flow-mediated dilation

Measurements of FMD were taken at baseline and p60, after cardiovascular parameters were measured. Subjects were placed in the supine position with the right arm in 90 degrees of abduction. The length between the acromion and olecranon was measured, and the midpoint was marked to identify the location for probe placement. Ultrasonographic images were obtained and analyzed in the same manner as echocardiographic images. A longitudinal image of the brachial artery and a Doppler image of brachial blood flow were recorded simultaneously in duplex mode using a 5-MHz linear probe. The insonation angle was set at <65 degrees to the direction of blood flow (Picot and Embree, 1994). The sample volume gate was adjusted to cover the width of the vessel. Images were recorded for 1 min at rest. Next, a rapid cuff inflator (E-20, D. E. Hokanson, Bellevue, WA, United States) on the right forearm was inflated to 250 mmHg for 5 min to occlude blood flow in the forearm. After reperfusion, there was a recovery period of 2 min. Vessel diameter and blood flow velocity were recorded continuously from 1 min before ischemia to 2 min after reperfusion.

Vessel diameter was measured every 10 s. Three measurements were made for each image and the mean value was calculated. The mean blood velocity (MBV) of one cardiac cycle was measured at the same time as diameter measurements were taken. The percent increase in vessel diameter from baseline to maximum vessel diameter after reperfusion was defined as %FMD. Shear rate was calculated using the following formula: shear rate = 8 × vessel diameter/MBV (Parker et al., 2009). The shear rate area under the curve was calculated as the sum total of shear rates every 10 s from the onset of cuff deflation to the point of maximum vessel diameter. We excluded one subject from the analysis due to technical problems during the NNsubmax trial. The between-day coefficient of variation for vessel diameter was found to be 0.8% in the present study.

### Statistical Analysis

Data are presented as means ± standard deviation. The significance level was set at 0.05. We used SPSS version 21 (IBM Co., Armonk, NY, United States) for all statistical analyses. One-way (condition) ANOVA was used for saturation and exercise parameters. Two-way (condition × time) repeated-measures ANOVA was used to analyze time-dependent changes in cardiovascular parameters. For *post hoc* analysis, the Bonferroni method was used where significant values were found.

## Results

### Oxygen Saturation

We confirmed that SpO_2_ in HH conditions was 93.0 ± 1.4%, which was lower than that in NN (99.1 ± 0.7%) or NNsubmax conditions (99.0 ± 0.6%) (*p* < 0.05).

### Exercise Parameters

Recorded exercise parameters are shown in Table 1. Exercise time, an index of performance, was shorter in HH and NNsubmax conditions compared with NN (*p* < 0.05). There were no differences in 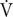
CO_2_mean, 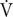
O_2_mean, 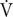
O_2_peak, and HRpeak between the conditions, although the difference in 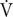
O_2_peak per kg body weight was close to statistical significance (*p* = 0.07). However, 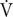
Emean was higher in HH than in the other two conditions (*p* < 0.05).

**Table 1:** Exercise parameters.

	NN	HH	NNsubmax	*p*-value
**Exercise time, min**	13.3 ± 2.6	10.2 ± 1.3^∗^	10.2 ± 1.3^∗^	*p* < 0.05
** 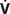 Emean, L/min**	99 ± 9	119 ± 13^∗†^	88 ± 8	*p* < 0.05
** 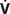 CO_2_mean, L/min**	3.06 ± 0.29	3.15 ± 0.26	2.88 ± 0.30	NS
** 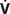 O_2_mean, L/min**	3.28 ± 0.35	3.14 ± 0.32	3.19 ± 0.33	NS
** 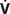 O_2_peak, L/min**	3.52 ± 0.37	3.27 ± 0.31	3.42 ± 0.32	NS
** 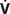 O_2_peak, mL/min/kg**	61.0 ± 3.7	56.2 ± 4.5	58.8 ± 2.8	*p* = 0.07
**HRpeak, beats/min**	190 ± 12	182 ± 5	180 ± 11	NS

*Data are presented as means ± standard deviation. Key: ^∗^p < 0.05, compared with NN; ^†^p < 0.05, compared with NNsubmax. NN, maximal exercise under normobaric normoxic conditions; HH, maximal exercise under hypobaric hypoxic conditions; NNsubmax, submaximal exercise under normobaric normoxic conditions; NS, non-significant;*
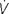
*Emean, mean minute ventilation during exercise;*
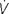
*CO_*2*_mean, mean carbon dioxide production during exercise;*
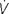
*O_*2*_mean, mean oxygen uptake during exercise;*
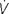
*O_2_peak, peak oxygen uptake; HRpeak, peak heart rate.*

### Post-exercise Hemodynamics

**FIGURE 1:**
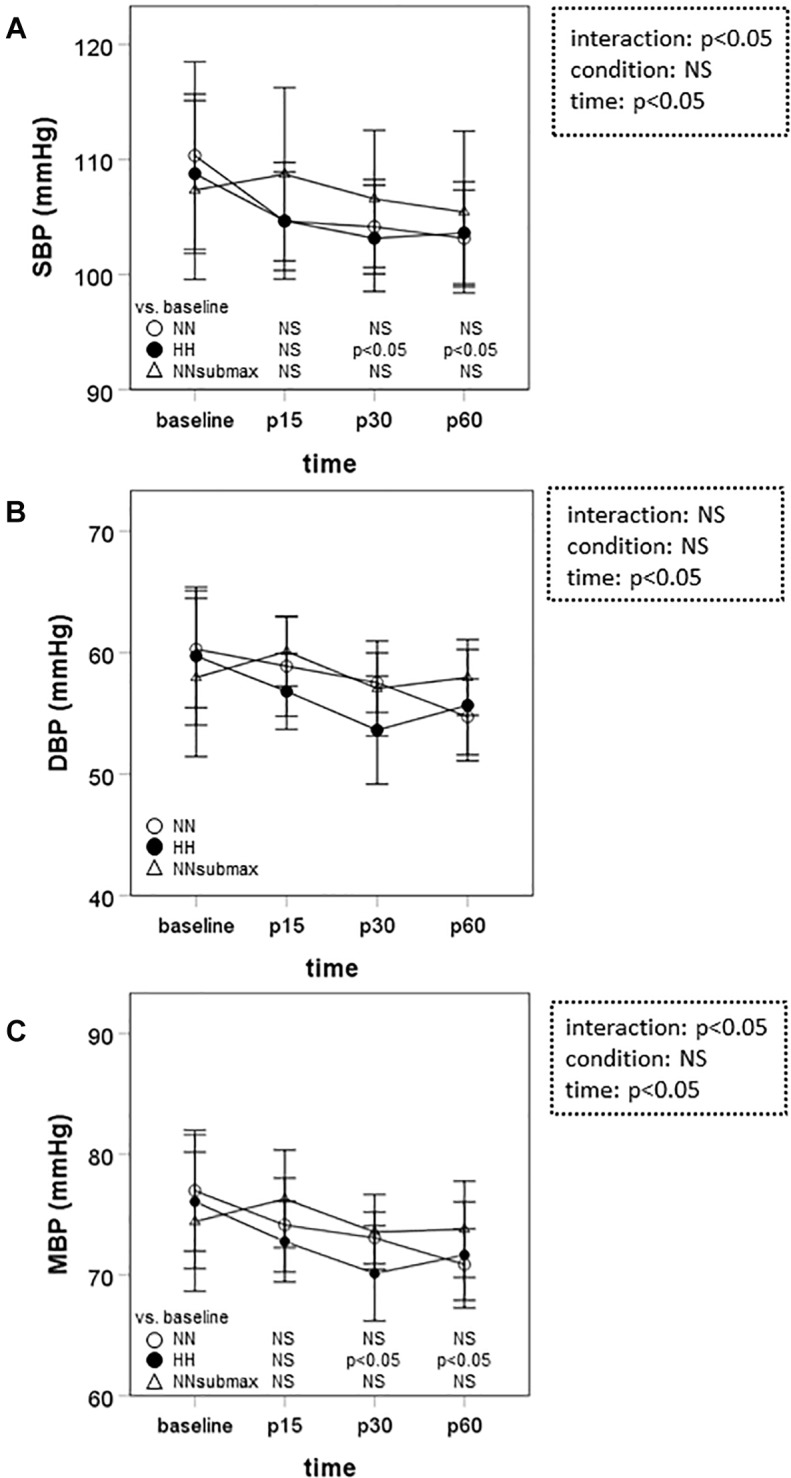
Time course measurements of blood pressure. Data are presented as means ± standard deviation. NN, maximal exercise under normobaric normoxic conditions; HH, maximal exercise under hypobaric hypoxic conditions; NNsubmax, submaximal exercise under normobaric normoxic conditions; p15, 15 min after exercise; p30, 30 min after exercise; p60, 60 min after exercise; NS, non-significant; SBP, systolic blood pressure; DBP, diastolic blood pressure; MBP, mean blood pressure.

**FIGURE 2:**
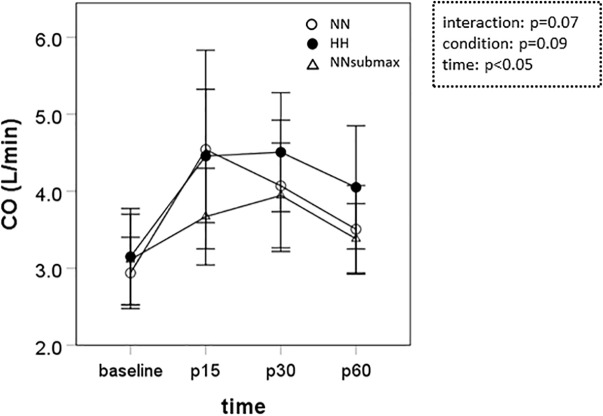
Time course measurements of cardiac output. Data are presented as means ± standard deviation. NN, maximal exercise under normobaric normoxic conditions; HH, maximal exercise under hypobaric hypoxic conditions; NNsubmax, submaximal exercise under normobaric normoxic conditions; p15, 15 min after exercise; p30, 30 min after exercise; p60, 60 min after exercise; NS, non-significant; CO, cardiac output.

Time course measurements for blood pressures are shown in Figure 1A–C. Both SBP and MBP were lower at p30 and p60 than baseline in HH conditions (*p* < 0.05). Regarding DBP, no difference was observed between the trials. The condition-related differences in HR, as assessed by ANOVA, were not statistically significant between HH and NNsubmax (*p* = 0.06) (Table 2). Neither stroke volume (Table 2) nor CO (Figure 2) showed any significant differences between the conditions.

**Table 2:** Values and results of analysis of variance of cardiac function before and after exercise.

	Condition	Baseline	p15	p30	p60	*p*-value
**HR, beats/min**	**NN**	49 ± 7	73 ± 13	64 ± 14	58 ± 9	Interaction: NS
	**HH**	50 ± 9	75 ± 9	67 ± 10	60 ± 11	Condition: *p* < 0.05
	**NNsubmax**	48 ± 10	67 ± 11	60 ± 11	55 ± 8	Time: *p* < 0.05
**SV, mL**	**NN**	60 ± 12	62 ± 13	64 ± 7	61 ± 9	Interaction: *p* = 0.05
	**HH**	64 ± 11	59 ± 12	67 ± 11	68 ± 11	Condition: NS
	**NNsubmax**	65 ± 13	55 ± 8	66 ± 6	62 ± 6	Time: *p* < 0.05

*Data are presented as means ± standard deviation. NN, maximal exercise under normobaric normoxic conditions; HH, maximal exercise under hypobaric hypoxic conditions; NNsubmax, submaximal exercise under normobaric normoxic conditions; p15, 15 min after exercise; p30, 30 min after exercise; p60, 60 min after exercise; NS, non-significant; HR, heart rate; SV, stroke volume.*

**FIGURE 3:**
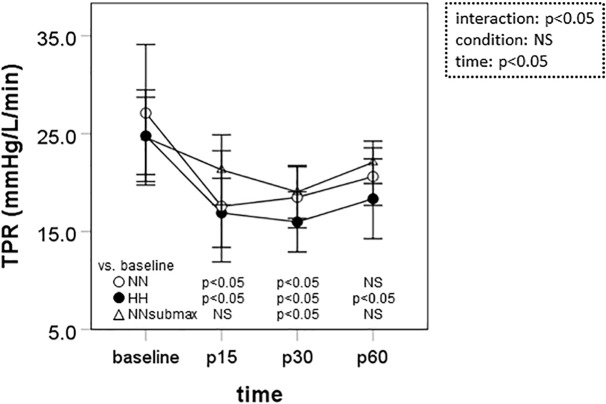
Time course measurements of total peripheral resistance. Data are presented as means ± standard deviation. NN, maximal exercise under normobaric normoxic conditions; HH, maximal exercise under hypobaric hypoxic conditions; NNsubmax, submaximal exercise under normobaric normoxic conditions; p15, 15 min after exercise; p30, 30 min after exercise; p60, 60 min after exercise; NS, non-significant; TPR, total peripheral resistance.

Time course measurements of TPR are shown in Figure 3. Under HH conditions, TPR was below baseline until the end of the measurement period (*p* < 0.05). In NN and NNsubmax conditions, TPR was only lower than baseline until p30 (*p* < 0.05).

The FMD parameters are shown in Table 3. The pre-occlusion diameter at p60 was higher in HH compared with NNsubmax conditions (*p* < 0.05). There were no other significant differences between baseline and p60 under any of the studied conditions except for the peak diameter (*p* < 0.05).

## Discussion

We hypothesized that mild hypobaric hypoxia enhances sustained post-exercise vasodilation in endurance athletes. This is the first study to investigate the effects of mild hypoxia on vasodilation after intense endurance exercise. The differences in SBP, MBP, and TPR responses to exercise under the different conditions of this study suggest that the reduction in systemic vascular resistance after intensive endurance exercise persists longer after exercise in mild HH conditions despite the lower exercise volume.

**Table 3:** Values and results of analysis of variance of flow-mediated dilation.

	Condition	Baseline	p60	*p*-value
**Brachial artery pre-occlusion diameter, mm**	**NN (*n* = 7)**	3.8 ± 0.2	3.9 ± 0.3	Interaction: *p* < 0.05
	**HH (*n* = 7)**	3.9 ± 0.2	3.9 ± 0.3	Condition: NS
	**NNsubmax (*n* = 6)**	3.9 ± 0.2	3.8 ± 0.3^∗^	Time: NS
**Brachial artery peak diameter, mm**	**NN (*n* = 7)**	4.1 ± 0.2	4.1 ± 0.2	Interaction: NS
	**HH (*n* = 7)**	4.1 ± 0.3	4.2 ± 0.2	Condition: NS
	**NNsubmax (*n* = 6)**	4.0 ± 0.3	4.1 ± 0.3	Time: *p* < 0.05
**FMD, %**	**NN (*n* = 7)**	5.6 ± 3.5	4.0 ± 4.5	Interaction: NS
	**HH (*n* = 7)**	5.7 ± 2.2	8.6 ± 4.2	Condition: NS
	**NNsubmax (*n* = 6)**	4.0 ± 2.0	7.5 ± 1.2	Time: NS
**SRauc, AU**	**NN (*n* = 7)**	588 ± 139	597 ± 147	Interaction: NS
	**HH (*n* = 7)**	498 ± 158	616 ± 229	Condition: NS
	**NNsubmax (*n* = 6)**	548 ± 221	464 ± 79	Time: NS
**FMD/SRauc**	**NN (*n* = 7)**	0.011 ± 0.008	0.006 ± 0.007	Interaction: NS
	**HH (*n* = 7)**	0.012 ± 0.005	0.016 ± 0.009	Condition: NS
	**NNsubmax (*n* = 6)**	0.014 ± 0.021	0.017 ± 0.004	Time: NS

*Data are presented as means ± standard deviation. Key: ^∗^*p* < 0.05, compared with HH. NN, maximal exercise under normobaric normoxic conditions; HH, maximal exercise under hypobaric hypoxic conditions; NNsubmax, submaximal exercise under normobaric normoxic conditions; p60, 60 min after exercise; NS, non-significant; FMD, flow-mediated dilation; SRauc, shear rate area under the curve; AU, arbitrary unit. We excluded one subject from the analysis due to technical problems during the NNsubmax trial.*

Systemic vascular resistance is controlled by neural and local factors (Pescatello et al., 2004). Sympathetic activity is a neural factor that increases systemic vascular resistance. In general, sympathetic activity increases with short-term exposure to hypoxia via chemical receptors (Smith and Muenter, 2000). In the conditions of the present study, however, hypoxia-induced sympathetic vasoconstriction may have been overcome by compensatory vasodilation. With regards to local factors, hypoxia augments release of the endothelium-derived vasodilators adenosine (Leuenberger et al., 1999), prostaglandin (Messina et al., 1992), and nitric oxide (NO) (Casey et al., 2010). Hypoxia-induced expression of endothelial NO synthase mRNA (Shaul et al., 1995; Le Cras et al., 1998; Xiao et al., 2001) may be implicated in hypoxia-induced vascular responses because receptors for hypoxia-inducible factor, a transcriptional regulator, are present in the endothelial NO synthase promoter area (Coulet et al., 2003). However, brachial artery FMD, which represents an index of NO-mediated vasodilation, was not affected by hypoxia in the present study. Katayama et al. (2016) reported that reactive hyperemia in response to exercise was not enhanced in the inactive limb of subjects breathing a hypoxic gas mixture (12.0% fraction of inspired oxygen, FiO_2_) compared to a normoxic gas mixture (21.0% FiO_2_), although it was enhanced in the active limb (Katayama et al., 2013). We measured FMD in the upper arm, which was active, although secondary to the lower limbs. Effects of hypoxia on post-exercise FMD might have been identified if FMD had been measured in the arteries of the leg. On the other hand, it is possible that local factors other than NO contribute to the impact of hypoxia on TPR. A previous study showed that sustained post-exercise vasodilation is dependent on activation of the histamine H1 and H2 receptors (McCord and Halliwill, 2006). However, it remains unclear whether the histamine response is modified by hypoxia. Further studies are needed to understand the details of the mechanisms underlying the prolonged post-exercise decrease in systemic vascular resistance that was observed in HH conditions in this study.

Most of the subjects of previous studies on hypoxic vasodilation were sedentary young individuals (Casey et al., 2010; Katayama et al., 2013). In contrast, we showed that competitive runners exhibited sustained post-exercise decreases in systemic vascular resistance after exercise in mild HH conditions. The expected angiogenesis of the microvasculature, which may be related to post-exercise sustained vasodilation, is more important in such endurance athletes due to their requirement for increased aerobic capacity compared with sedentary individuals. In addition, HH conditions may be more beneficial than conditions of normobaric hypoxia for endurance-trained athletes who compete in high-speed running, because the reduced air density modifies the air resistance, and facilitates high-speed movements (Ward-Smith, 1984). However, normobaric hypoxia may have other benefits for endurance athletes (Millet et al., 2012).

Our findings could contribute to the development of a new training method for athletes. Furthermore, elderly or hypertensive subjects who need to improve their vascular function may benefit from hypoxic exercise. The combination of hypoxic stimuli and exercise improves several parameters of vascular function, including reduced blood pressure, which are pertinent for the reduction of cardiovascular risks (Millet et al., 2016). Therefore, we propose that altitude exercise has possible clinical applications to various populations, although further studies are needed for this potential to be realized. First, although we have demonstrated that the post-exercise reduction in SBP, MBP, and TPR persist for longer after exercise in HH conditions, we did not observe the recovery of baseline for these parameters. Future studies of post-exercise hemodynamics with increased observation periods could provide important insight. Second, the effects of hypoxia on vasculature depend on the hypoxic level, as indicated by the finding that mortality from stroke decreases with increasing altitude of the place of residence (12% per 1,000 m) (Faeh et al., 2009). The effects of low- and high-intensity hypoxia may differ with regards to the mechanisms involved in post-exercise vasodilation. Identifying the optimal altitude for enhancement of post-exercise vasodilation would be highly significant for athletes and clinicians.

The present study had some limitations. First, we examined only acute-vessel responses to a single bout of endurance exercise. A long-term intervention study is needed to ascertain whether the sustained vasodilation after exercise under conditions of hypoxia contributes to growth and remodeling of the microvasculature. Second, since we studied young male collegiate runners, we cannot generalize the present findings to the entire population. Third, the sample size was relatively small, and we found that some differences in measurements did not reach statistically significant level.

## Conclusion

Our findings suggest that reductions in systemic vascular resistance induced by endurance exercise might persist longer under mild HH conditions (equivalent to 2,200 m above sea level), even when the absolute exercise volume is reduced, and compared with NN conditions (equivalent to sea level). Mild HH and post-exercise hemodynamics seem to have additive effects in young runners when compared with NN conditions.

## Ethics Statement

This study was carried out in accordance with the recommendations of Ethical Guidelines for Medical and Health Research Involving Human Subjects, the Ministry of Education, Culture, Sports, Science and Technology of Japan with written informed consent from all subjects. All subjects gave written informed consent in accordance with the Declaration of Helsinki. The protocol was approved by the Ethics Committee of the Japan Institute of Sports Sciences.

## Author Contributions

YS contributed to the entire process of the work including the design of the study, acquisition, analysis, interpretation of the data, and writing of the manuscript. MN and KE contributed to the acquisition and interpretation of the data. TO contributed to the interpretation of data and the critical repeated revisions of the manuscript. All authors contributed to manuscript revision and have read and approved the submitted version.

## Conflict of Interest Statement

The authors declare that the research was conducted in the absence of any commercial or financial relationships that could be construed as a potential conflict of interest.
